# Essential reporting items within a law enforcement recruit injury and physical performance database: A modified Delphi study

**DOI:** 10.1016/j.jsampl.2023.100035

**Published:** 2023-09-02

**Authors:** Myles C. Murphy, Simone Radavelli-Bagatini, Garth Allen, Nicolas H. Hart, Andrea B. Mosler

**Affiliations:** a Nutrition & Health Innovation Research Institute, School of Medical and Health Sciences, Edith Cowan University, Joondalup, Western Australia, Australia; b School of Health Sciences and Physiotherapy, The University of Notre Dame Australia, Fremantle, Western Australia, Australia; c Western Australian Police Academy, Western Australian Police Force, Joondalup, Western Australia, Australia; d Exercise Medicine Research Institute, School of Medical and Health Sciences, Edith Cowan University, Joondalup, Western Australia, Australia; e Human Performance Research Centre, School of Sport, Exercise, And Rehabilitation, Faculty of Health, University of Technology Sydney, Sydney, New South Wales, Australia; f Caring Futures Institute, College of Nursing and Health Science, Flinders University, Adelaide, South Australia, Australia; g Institute for Health Research, The University of Notre Dame Australia, Fremantle, Western Australia, Australia; h School of Nursing, Faculty of Health, Queensland University of Technology, Brisbane, Queensland, Australia; i La Trobe Sport and Exercise Medicine Research Centre, La Trobe University, Bundoora, Victoria, Australia

**Keywords:** Police, Physical performance, Injury, Tactical operator, Recruit

## Abstract

**Objective:**

Collate the perceptions and experience of relevant key stakeholders to develop reporting guidelines for epidemiological injury and physical performance data within law enforcement agencies recruit training programs.

**Design:**

An augmented Delphi consensus process.

**Methods:**

Initial item generation occurred via online, one-on-one, semi-structured interviews, and followed by one survey round. Items generated from interviews were categorised within three main categories: i) Demographic data, ii) Injury data, and iii) Physical performance data. Participants represented one-of-six target groups: Police officers; Police physical training staff; Police occupational health and safety staff; Elite sport high performance staff; Military high-performance staff; Physical activity injury epidemiologists.

**Results:**

A total of 15 representatives (53% women) from six stakeholder groups were included. Other than responses directly related to item generation, three main themes emerged from round one: i) recruits are not likely to report all data being requested truthfully, ii) data that is recorded must be acted upon, and iii) body fat assessments should not be included in this population with focus instead being placed on performance. Three separate reporting databases were generated.

**Conclusion:**

Our study established clear demographic, mental health/physical injury, and physical performance data to be collected in a law enforcement recruit training program for injury surveillance and performance monitoring. Furthermore, we identified several items that were classified as relevant, but unlikely to be reported truthfully. These items can help inform current practice and assist clinicians to determine the trustfulness of information received by patients when working within law enforcement environments.

## 1 Introduction

Musculoskeletal conditions are the third largest health burden for Australia's age standardised disability-adjusted life years [1] and are responsible for the majority of Australia's health expenditure [2]. Operational police officers experience a larger proportion of all-cause injury than civilian occupations, resulting in a substantial healthcare burden [3]. Medical costs associated with operational police officer time-loss injuries in Victoria alone amounted to ∼$19 million AUD from 2002 to 2012 [3].

Limited epidemiological data on injuries and risk factors for injury exist for Police Force recruits [4]. Similarly, there are few published reports of injury prevention strategies that have been conducted in this population. Data from a 2022 systematic review showed that less than ten studies worldwide reported law enforcement recruit injury epidemiology [4]. Furthermore, the injury data in the law enforcement studies was of low quality, representing retrospective analyses of data collected within routine practice, and few studies reporting injury detail such as body region or type [[4], [5], [6], [7], [8], [9]]. The limited data in law enforcement recruits sharply contrasts with military recruit populations, where a recent systematic review of military recruit injury epidemiology identified over 41 studies published on the field since 2000 [10].

In 2022, the most comprehensive analyses of law enforcement recruit injury epidemiology to date were published, using data from a single jurisdiction. However, these data were limited by the fact that they were collected by law enforcement agencies via routine practice.[11,12] For example, almost 30% of injuries were unable to be classified to an injury region, which has significant implications to developing effective injury prevention strategies. Unlike sporting [13] and military [14] injury surveillance systems that have clear recommendations on the data that should be captured and data capture methodology, standardised reporting requirements and methodology for law enforcement surveillance systems does not exist.

Although the Translating Research into Injury Prevention Practice framework proposes a six-step protocol to target injury prevention [15], the implementation of this framework is currently limited in the law enforcement setting. A major barrier is the lack of existing recommendations and standard practice on injury and exposure surveillance required to determine injury patterns and aetiology or assess the effectiveness of an injury prevention intervention. The development of injury reporting guidelines would allow policy makers within law enforcement agencies to amend current injury surveillance systems and collect data that is meaningful to the recruit and agency as well as more applicable to injury prevention research. The burden on both the recruit and the organisation must also be considered when collecting data [16].

Our objectives we to collate the perceptions and experience of relevant key stakeholders to develop reporting guidelines for epidemiological injury and physical performance data within law enforcement agencies recruit training programs.

## 2 Methods

An augmented Delphi consensus process [17] was conducted between the 1st July 2022 and the 30th November 2022, with initial item generation occurring via online, one-on-one, semi-structured interviews and was followed by one round of survey responses.

This study included the input of experts with knowledge related to a specific domain considered important by both the research team and the Police Force for the development of an injury and physical performance database of relevance to Police Force recruits. To achieve diversity of participant expertise, we recruited 1–4 participants from each of the following expert groups to cover all relevant domains of interest.

1. Police Force operational officers
2. Police Force physical training staff
3. Police Force occupational health and safety staff
4. Elite sport high performance sport science and sports medicine staff
5. Military high-performance sport science and sports medicine staff
6. Physical activity injury epidemiologists

Participants were identified via the research team (academic and industry) networks, as well as through google scholar searches for relevant expertise. Participants were then invited to participate via email. We included a sample of diverse genders and used purposive sampling to select participants from the research teams’ networks to ensure we included appropriate numbers and a range of participants across each of the six domains of content expertise.

Recommended sample sizes for database generation do not exist currently. Therefore, the COnsensus-based Standards for the selection of health Measurement INstruments (COSMIN) guidelines were used to provide sample sizes for establishing content validity that equate to inadequate, doubtful, adequate or very good quality. For the purposes of this study, Police Force staff (i.e., groups 1 to 3) were consumers, and external experts (groups 4 to 6) were considered professionals. The COSMIN guidelines state that 4 to 6 participants within the consumer group and 4 to 6 participants within the professional group are adequate for qualitative research, and greater than seven participants is very good [18].

Round one of the Delphi consensus process involved semi-structured one-on-one online interviews (approx. 30–45 ​min in duration), conducted by one member of the research team with qualitative research experience (MM) [[19], [20]]. The personal characteristics of the interviewer are described in Appendix A. A cognitive interviewing technique [21] was employed for the first four interviews with participants asked if they understood the questions and task and asked to provide suggestions on how the interview process might be improved. Following this, small changes were made in accordance with expert suggestions to the structure and flow of the interview to generate improved responses and improve the experience for participants.

Items generated from interviews were categorised within three main categories: i) Demographic data: including measures such as age, ethnicity, and injury history, ii) Injury data: including measures such as injury region, type, time-loss, and mechanism of injury and, iii) Physical performance data: including measures such as training load, cardiorespiratory and fitness testing, and wellness data.

The aim of the first round was item generation, where participants were provided a preliminary list of items outlined in Appendix B (based on existing Police Force systematic reviews [4], consensus statements [13] and original research [[5], [6], [7], [8], [9], [11], [12], [22], [23]]) and given time to review it. Interviews were conducted online via Microsoft Teams and audio recorded (with participant consent) in full. Participants were explained the study background, aims, and methodology. The interviewer prompted participants to propose any additional items and then provide a rationale for their inclusion. The item list was edited in real time, so subsequent participants were able to view inclusions from other experts. Participants were also asked to provide their input on existing items and whether the item should stay or be removed. However, even if participants requested an item be removed, we did not remove items during this round to get as diverse a list of items as possible. Saturation was determined by the lack of new themes emerging from additional interviews [24]. New interviews were conducted until three consecutive interviews resulted in no additional items included, suggesting that saturation had been reached. Audio files were transcribed using DeScript software and cross-checked using the initial interview recording.

One researcher (MM) coded all data using QSR NVIVO (Version 12.6.1.970) and senior members of the research team (NHH and ABM) provided oversight and guidance. A template thematic analysis approach was used whereby the coding structure was guided by the initial items and were built on during the coding phase. This generated a greatly expanded list of items (presented in Round Two).

Round two of the modified Delphi consensus process was completed via an online survey (Qualtrics) and involved participants providing feedback on all items generated for inclusion in the database from round one. Using a similar process to testing the content validity of a patient reported outcome measure [25], the items were assessed by participants by assigning scores for the *relevance, comprehensiveness*, and *comprehensibility* of each item [18]. Additionally, based on a theme that emerged in round one, participants were also required to score the expected ‘*truthfulness*’ of the information if the data was self-reported by recruits (e.g., self-reported wellness or alcohol consumption). The *relevance* of every item and the *truthfulness* of every recruit-reported item from round one was scored by all participants, as well as the overall *comprehensiveness* and *comprehensibility* of the database. All scoring was performed on a five-point Likert scale (strongly disagree, disagree, neither agree nor disagree, agree, strongly agree). The option for providing additional comments was given for each item.

Following completion of round two, one study author (MM) exported the Qualtrics data to SPSS statistics package (Version 28.0.1.0.142). The frequency of responses for all items was calculated with results presented as a percentage in favour of inclusion of that item. An a-priori cut-off of 75% was established by the research team as the cut-off for inclusion. Items that did not achieve a consensus of 75% or greater for *relevance* were removed from the list of recommended items. Items that did not achieve a consensus of 75% or greater for *truthfulness* were also removed from the list of recommended items. This percent agreement was based upon reducing the burden of data entry/collection by staff by removing items not deemed to be useful (e.g., not relevant or likely to be truthful).

A third round of consensus was planned that would be identical to phase two, which included items judged as relevant but not comprehensive and were heavily amended from phase two. Participants would again judge the *comprehensibility, comprehensiveness, relevance and truthfulness.*

## 3 Results

A total of 15 representatives from six target groups (Table 1) were included in this study. Participants had a balanced gender split (8/15, 53%) and represented various areas of Australia: Western Australia (9/15, 60%); New South Wales (2/15, 13.3%); South Australia (2/15, 13.3%); Australian Capital Territory (1/15, 6.7%); Victoria (1/15, 6.7%). Three participants reported being unavailable at the time of round one interviews but expressed a willingness to participate and were therefore included in round two only.

**Table 1:** Characteristics of participants.

Representative Group	Domain	Gender	Australian geographical location
Consumer	Western Australian Police Force physical training staff	Man	Western Australia
Consumer	Western Australian Police Force physical training staff	Man	Western Australia
Consumer	Western Australian Police Force occupational health and safety staff	Woman	Western Australia
Consumer	Operational Western Australian Police Force officer	Man	Western Australia
Consumer	Operational Western Australian Police Force officer	Woman	Western Australia (Remote)
Consumer	Operational Western Australian Police Force officer	Woman	Western Australia
Professional	Physical activity injury epidemiologists	Woman	Victoria
Professional	Physical activity injury epidemiologists^a^	Man	New South Wales
Professional	Elite sport high performance staff	Man	New South Wales
Professional	Elite sport high performance staff	Woman	Australian Capital Territory
Professional	Elite sport high performance staff	Woman	South Australia
Professional	Elite sport high performance staff	Man	Western Australia
Professional	Military high-performance staff^a^	Man	Western Australia
Professional	Military high-performance staff^a^	Woman	Western Australia
Professional	Military high-performance staff	Woman	South Australia

a Did not complete the round one interview but completed the round two survey.

Participants provided a number of responses for specific item generation. Furthermore, three themes emerged from our item generation process in round one: i) potential recruits are not likely to report all data being requested truthfully (predominantly developed from the opinions of Police Force officers and staff), ii) data that is recorded must be acted upon, and iii) body fat assessments should not be included in this population with focus instead being placed on performance. Furthermore, a number of participants included specific screening tests they felt should be included within a performance database, however the authors agreed that selecting specific tests was beyond the scope of this study and therefore, any specific tests identified by participants were not included in the overall results. These suggestions were instead grouped under screening tests (e.g., ankle range of motion) and physical performance tests (e.g., beep test).

### 3.1 Demographic data

Twenty-three items (n ​= ​23) were generated within round one and assessed for consensus in round two (Table 2). Four items (n ​= ​4) did not achieve consensus for *relevance*: body mass index; alcohol intake; education level; economic income bracket. Body mass index was scored poorly as participants stated it should just be calculated from height and weight, not recorded as its own item. Alcohol intake, education level, and income bracket were scored poorly due to concerns related to how the data would be used.

**Table 2:** Content validity of demographic items.

Surveillance system item	Relevance consensus (%)	Truthfulness consensus (%)
Name	85%	
Age (years)	100%	
Gender (M, W, NB, PNTS)	100%	
Ethnicity	92%	
Height (m)	92%	
Weight (kg)	92%	
Body Mass Index^a^	54%	
Occupational History		
Physically active or sedentary (yes/no)ˆ	77%	62%
Previous military/paramilitary role (yes/no)	85%	100%
Previous health/fitness position (yes/no)	77%	92%
Recruit medical history (open)ˆ	100%	54%
Family medical history (open)ˆ	77%	54%
Recruit musculoskeletal injury history		
Injuries with medical consult (number and diagnosis)ˆ	100%	46%
Time-loss (number and diagnosis)ˆ	85%	46%
Required hospitalisation/surgery (number and diagnosis)	85%	77%
Physical activity levels		
Pre-academy application (exercise type and session per week)	92%	77%
Post-academy application (exercise type and session per week)	92%	85%
Depression and anxiety symptom screen (score)	92%	77%
Recruit alcohol intake (standard drinks)â	69%	23%
Recruit medications (type and dosage)ˆ	77%	54%
Recruit smoking status, including vaping (yes/no)ˆ	85%	31%
Level of education (Year 8, Year 10, Year 12, diploma, bachelor degree, masters degree, doctoral degree)^a^	54%	93%
Income bracket (Dollars: 0–18,200; 18,201–45,000; 45,001–120,000; 120,001–180,000; 180,001 and over)â	46%	69%

Legend: M ​= ​man, W ​= ​woman, NB ​= ​non-binary, PNTS ​= ​prefer not to say, m ​= ​metres, kg ​= ​kilograms.

a Did not achieve consensus for *relevance, ˆ* did not achieve consensus for truthfulness.

Nine items (n ​= ​9) did not achieve consensus for *truthfulness*: occupational history (physically active or sedentary); medical history; family medical history; musculoskeletal injury history (medical attention and time-loss); alcohol intake; medications; smoking status; income bracket. Occupational history (physically active or sedentary), medical history, family medical history and musculoskeletal injury history were scored poorly due to concerns recruits would feel these data may impact their career. Alcohol intake, medications, smoking status and income bracket were scored poorly due to fear of being excluded from future training if answered truthfully.

The final list of included items, with linked validation, is presented within Appendix C as a resource freely available for all law enforcement agencies to utilise.

### 3.2 Injury data

Twenty-one mental health and injury surveillance items (n ​= ​21) were generated within round one and assessed for consensus in round two (Table 3). All injury items achieved consensus. However, some key themes for general consideration when interpreting injury data were recorded by participants.

**Table 3:** Content validity of mental health and injury surveillance items.

Surveillance system item	Relevance Consensus (%)
Physical injury/mental health occurrence (yes)	92%
Injury diagnosis (doctor or physiotherapist provided)	100%
Injury laterality (left, right, both sides, not applicable)	100%
Injury acuity (acute, chronic, acute on chronic, unsure, not applicable)	100%
Injury severity (all, medical attention, time-loss, absenteeism)	100%
When injury occurred (days into training)	100%
Duration injury required modified duties (days)	92%
Duration injury required in absenteeism (days)	92%
Injury (new, subsequent, recurrent)	100%
Physical injury region (as per IOC guidelines)	100%
Physical injury type (as per IOC guidelines)	92%
Mechanism of injury	
Non-contact, contact (with a person), contact (with an object)	92%
Within 60 ​min of sitting (yes/no)	92%
Did the injury follow 1-week of no physical training (yes/no)	92%
Activity of injury (Industry specified responses)	100%
Injury occurred on deployment (yes/no)	100%
Injury occurred performing a hazardous activity (yes/no)	100%
Environmental factors (open)	92%
Injury resulted in missing operational skills training (yes/no)	100%
What rehabilitation requirements and modifications are needed (open)	92%
Has appropriate healthcare referrals been initiated (yes/no)	100%

Legend: IOC= International Olympic Committee.

Firstly, participants stated that recruits are likely to underreport injuries and mental health conditions, due to potential restrictions these may place upon their capacity to work operationally with only 38% of respondents stating they agree recruits would accurately report injuries. Secondly, participants reported that the accuracy of the injury region/type is only going to be as accurate as what is recorded by the health care provider.

The final list of included items, with linked validation, is presented in Appendix D as a freely available resource for all law enforcement agencies to utilise.

### 3.3 Physical performance data

Thirteen items (n ​= ​13) were generated in round one and assessed for consensus in round two (Table 4). Six items did not achieve consensus for *relevance*: training soreness; fatigue; food diary; waist circumference; skinfolds and dual x-ray absorptiometry for body fat percentage assessment.

**Table 4:** Content validity of physical performance items.

Surveillance system item	Relevance consensus (%)	Truthfulness consensus (%)
Training load (time spent physically active)	85%	77%
Training load (time spent training outside the academy)^b^	85%	69%
Training soreness^a^^.^^b^	62%	54%
End of day fatigue^a^^.^^b^	62%	38%
General wellbeing	85%	92%
Sleep quality	85%	85%
Sleep duration	77%	77%
Coping^b^	85%	54%
Academy expectations being met	77%	85%
48-h food diary^a^^.^^b^	58%	54%
Waist circumference^a^	69%	
Skinfolds to assess body fat percentage^a^	39%	
Dual x-ray absorptiometry for body fat percentage^a^	54%	

a Did not achieve consensus for *relevance*.

b did not achieve consensus for truthfulness.

Training soreness was scored poorly as this is, to some degree, an expected result from training and unlikely to be actioned. End of day fatigue was scored poorly as participants suggested sleep quality is a better marker of recovery. The food diary, waist circumference, skinfolds, and dual x-ray absorptiometry to assess body fat percentage were all scored poorly due to the potential negative impacts they can have on recruits’ mental health. Instead, participants suggested these items might provide good value, but should be used on a case-by-case basis, as opposed to being introduced into a global surveillance system.

Five items did not achieve consensus for *truthfulness*: training load external to the academy; training soreness; end of day fatigue; coping; food diary. Participants reported training loads external to the academy are likely to be under- or over-reported based on the recruit. Similarly, to training loads, participants reported that soreness and fatigue are likely to be inaccurate and participants felt a food-diary is unlikely to be accurate as recruits will want to appear healthier than they are. Finally, whilst coping was reportedly likely to be inaccurate, participants (particularly operational officers) reported this is likely to be valuable as this may be a simple avenue for someone to ask for help and commence a conversation.

One key theme emerged from expert feedback on physical performance surveillance. Participants reported that whilst it can be tempting to better monitor training load, and have a measure of exposure, this should only be done if the resources are available to implement changes based on the collected data. Otherwise, this process is only likely to be burdensome.

A third round of consensus was planned similar to round two. However, the feedback within the comments that related to all items presented were not related to the *comprehensibility* or *comprehensiveness* of the database. Instead, commentary related to why participants voted a particular item as *not relevant or not truthful.* Therefore, no items required a further consensus round.

## 4 Discussion

Our modified Delphi study included 12 semi-structured interview responses followed by 14 survey responses and identified 19 demographic items, 21 mental health/physical injury items, and 7 physical performance items that should be included in a law enforcement recruit training program database. Furthermore, our study identified several additional items of relevance, but were reported as being unlikely to be answered truthfully. Our recommendations are translatable across all Australian Police Force jurisdictions given the similarities in recruit training structure, however their generalisability internationally is unknown.

Law enforcement recruits undergo strenuous physical training [11], are exposed to unique stressors (e.g., scaling walls and jumping fences) [12], and as a profession, have a higher injury incidence rate than would be expected for a civilian occupation [3]. Therefore, it is the prerogative of law enforcement agencies to implement strategies to maximise performance during training and minimise injury risk strategies to achieve these aims. These must be informed by accurate and relevant data to be effective. However, no recommendations exist for what data law enforcement agencies should be collecting. This differs substantially to sporting and military organisations, who have clear guidelines on what and how data should be collected within physical training programs [13,14]. Consistent recording of injury data (e.g., using international Olympic committee recommendations [13]) across agencies may be useful, and further research to develop law enforcement specific recommendations are needed.

Our study highlights specific information which both consumers and experts identified as relevant and likely to be truthfully answered by law enforcement recruits. We recommend that these items should be collected at the commencement of, and where applicable during, the law enforcement recruit training program. One key theme raised by participants was their clear concerns over what recorded data may be used for. This was the primary rationale to exclude suggested items as alcohol intake and some elements of injury history. In an elite athlete population, there is a recognised fear of accurately reporting data as it may result in some form of consequence, either to selection or socially [19]. Participants also felt that self-reported injury history is likely to be inaccurate due to recall-biases [26]. Therefore, we consider it vital that staff collecting such demographic data provide transparency to why these data are being collected, its relevance, and how the information will be used and protected, as this may improve reporting truthfulness. We consider this especially important in a population who must be accountable for integrity.

Participants stated that under-reporting of injury is highly likely, particularly for minor injuries which are likely to have poor recall and is a concern with past injury history typically being the strongest risk factor for recurrent and subsequent injury.^27.28^ This premise is supported by Merrick et al., 2022 who demonstrated that very mild and mild injuries made up the smallest proportion of a law enforcement injury database [11]. Under-reporting of injury in other population accountable for integrity (e.g., sport and military populations) is a well-documented limitation [[27], [28]], with underreporting of mental health concerns being particularly prevalent [28]. Therefore, in additional to collecting the data, it is essential that law enforcement agencies aim to provide a safe environment for recruits to feel comfortable in disclosing injury or mental health concerns to staff.

It was reported that ‘sensitive’ data such as smoking status may not be disclosed truthfully, for fear of how these data may be used. Therefore, previous studies that have assessed the influence of these variables (e.g., smoking status) on injury and physical performance [29], may need to be reconsidered. Further research into how these ‘sensitive’ variables may influence injury and performance will require further investigation with innovative methodology (e.g., data being reported to providers external to the Police Force) to get a more accurate reflection of the risk associated with these variables.

It was clear in the present study that participants felt many of the potential physical performance data that could be collected, are unnecessarily burdensome for police recruits and staff. It has been proposed in an athlete population that when collecting data, consideration of usefulness, validity and burden is essential [16]. Any physical performance measures must be informative and change practice, otherwise they provide no use and appear to just be collected for the sake of being collected. Any physical performance measures included must also be reliable and valid, to ensure the information can be trusted. Finally, the participants identified that the burden of both recruit and staff must be considered before including additional measures within a surveillance system. This has rarely been given consideration in other injury surveillance database developments.

## 5 Conclusion

Our study established clear demographic, mental health/physical injury, and physical performance data to be collected in a law enforcement recruit training program for injury surveillance and performance monitoring. Furthermore, we identified several items that were classified as relevant, but unlikely to be reported truthfully. These items can help inform current practice and assist clinicians to determine the trustfulness of information received by patients when working within law enforcement environments.

## Practical implications

• Law enforcement agencies employ sport scientists and healthcare providers who are responsible for performance and injuries during a recruits intensive physical training.
• Whilst injuries have a substantial burden for law enforcement recruits, standardised industry specific recommendations on epidemiological reporting do not exist.
• This study provides clear recommendations, developed in collaboration by research experts, law enforcement staff and consumers, on data essential to report in law enforcement report settings by sport and exercise staff.

## Author contributions

Myles Murphy, Garth Allen, Nicolas Hart and Andrea Mosler conceived the study concept and designed the methods. Myles Murphy collected all data and performed analysis. All authors contributed to manuscript preparation.

## Confirmation of ethical compliance

Ethical approval was obtained through the Edith Cowan University Human Research Ethics Committee (ID: 2022-03536-MURPHY) with research governance approval obtained through the WA Police Force Research Governance division. As a modified Delphi study, inclusive of qualitative data from one-on-one interviews, this study data will not be made freely available to ensure the anonymity of participants.

## Funding information

This research was funded via a collaborative research grant between the Western Australia Police Force and the Edith Cowan University School of Medical and Health Sciences (Grant ID: G1006360).

## Declaration of competing interest

Garth Allen is an employee of the Western Australian Police Force, but had no role in data collection, analysis or interpretation. All other authors have no relevant financial or non-financial interests to disclose.
