# Supplementary material for: Essential reporting items within a law enforcement recruit injury and physical performance database: A modified Delphi study
Source: JSAMS Plus. 2023 Sep 2;2:100035. doi: 10.1016/j.jsampl.2023.100035 (PMC13008423; doi:10.1016/j.jsampl.2023.100035)
Supplement: Multimedia component 1 [file mmc1.docx]

**Appendix A. Characteristics of interviewer**

| **Credentials** | **Occupation** | **Age** | **Gender** | **Ethnicity** | **Experience and Training** |
| --- | --- | --- | --- | --- | --- |
| Physiotherapist, PhD | University academic and clinical sport and exercise physiotherapist | 31 years | Man | White Australian | Has worked for greater than five years in clinical practice communicating with patients, including Police Force officers, and has published several research papers on law enforcement injury epidemiology. Finally, he has completed several qualitative research projects and is leading the development of an international consensus team creating a new outcome measure for Achilles tendinopathy |

**Appendix B. Items included for discussion within round one**

| Demographics | Age (Years) |
| --- | --- |
|  | Sex (M/F/Prefer not to say) |
|  | Ethnicity (Freehand) |
|  | Height (cm) |
|  | Weight (kg) |
| Injury | Diagnosis (As per clinical notes) |
|  | Definition: Medical attention, time-loss, absenteeism, other |
|  | Region (set categories)   - Foot - Ankle - Lower leg - Knee - Thigh - Hip/ groin - Lumbosacral - Thoracic spine - Trunk (abdomen) - Trunk (chest) - Neck - Hand - Wrist - Elbow - Upper arm - Shoulder - Head - Multiple sites - Respiratory |
|  | Type (set categories)   - Bone (stress injury) - Bone (contusion) - Bone (fracture) - Cartilage, synovium or bursa - Infection - Ligament or joint capsule - Muscle or tendon - Nervous system or brain - Superficial tissue (skin) - Non-specific |
|  | Activity of injury (set categories)   - Physical Training - External to academy - Unknown - Operational skills training - Empty hand training - Other |
|  | Mechanism of injury (No set categories) |
|  | Time into training (days) |
|  | Time modified (days) |
|  | Missed Operational skills training (Yes/ No) |
| Physical Performance | Police specific physical performance measures   - Beep test (level) - Physical Performance Evaluation (time) - Max push ups (reps) - 5km Time Trial (seconds) |
|  | Time spent in physical training versus academic learning. (time) |
